# When Everyone Wins? Exploring Employee and Customer Preferences for No-Haggle Pricing

**DOI:** 10.3389/fpsyg.2018.01555

**Published:** 2018-09-06

**Authors:** Kevin M. Kniffin, Richard Reeves-Ellington, David S. Wilson

**Affiliations:** ^1^Dyson School of Applied Economics and Management, S. C. Johnson College of Business, Cornell University, Ithaca, NY, United States; ^2^Binghamton University – The State University of New York, Binghamton, NY, United States

**Keywords:** evolutionary psychology, fairness, employee satisfaction, pricing, customer satisfaction, prosocial

## Abstract

The organizational importance for interactions between frontline employees and customers has been examined in relation to dimensions such as climate or culture. In this article, we highlight the importance of pricing strategies – typically studied in relation to consumer preferences – for frontline employees. To do this, we apply an evolutionary perspective and present two complementary studies that focus on the relevance of price discipline in relation to employee attitudes and preferences. Focusing on the industry of new automobile sales since there is important firm-level pricing variation, Study 1 finds a faintly positive relationship among employee prosociality, customer satisfaction, and fixed or “no-haggle” pricing strategies. In Study 2, participants indicated a preference for working in environments that offered the same, non-disparate prices to all customers. While previous research has examined the relationships between employee and customer attitudes in relation to firm performance, our studies emphasize the role that pricing strategies can play as a mechanism in those relationships. Our studies illustrate the value of evolutionary frameworks for contemporary business problems.

## Introduction

Concern about one’s relative standing with respect to salary and consumption preferences has been closely studied with the benefit of evolutionary perspectives. For example, Frank (2001, 2012) has repeatedly highlighted broad preferences for relatively high amounts of salary or goods when compared with absolute levels or amounts. Obversely, Kuziemko et al. (2014) finding of “last-place aversion” is fitting since it shows that people appear especially averse to feeling as if they have the least amount of a given good when compared with others.

While the importance of relative – instead of absolute – fitness across evolutionary time (Wilson, 2004) helps make sense of such concerns, the patterns found by consumption research are even more sensible in an evolutionary light when considering the comparable findings reported from studies of non-human primates. Specifically, Brosnan and colleagues (e.g., Brosnan and De Waal, 2003; Brosnan et al., 2013) have found a tendency for individuals to react negatively if someone important to them (e.g., in a neighboring cage) gains more than they gain from a common attendant. Similarly but based on observations of non-human primates observing human interactions, Anderson et al. (2013) find evidence that non-human primates react negatively to people who they observe to be selfish or unhelpful in relation to other people.

Rather than suggesting that people tend to want to “keep up with the Joneses” (Frank, 1984; Luttmer, 2005; Kniffin, 2009; Norton, 2013), evidence indicates that it would be more precise to recognize that people (a) would like to be “ahead of” the Joneses and (b) especially do not want to be “last” among those who care about the Joneses. With respect to the dimension(s) on which people (or non-human primates) keep score (e.g., with the Joneses), it is clear that there exists a wide array of context-specific currencies for which people care about their relative wealth. During the Environment of Evolutionary Adaptedness (EEA) (e.g., Tooby and Cosmides, 1990), at least, it would have been adaptive for people to be concerned about their relative standing with respect to dimensions (e.g., goods or skills) that were positively associated with reproductive fitness and we know from the anthropological record that such currencies have included – in addition to money, where applicable – animal hides, turtles, and, more generally, gifts (e.g., Hawkes and Bliege Bird, 2002; Smith, 2004; Kruger, 2008).

In this paper, we consider the currency of getting or providing “a good deal” as a dimension on which people appear to be concerned as both buyers and sellers. Our interests build upon evolutionary studies that focus on the marketing and consumption of specific product types (e.g., Hantula, 2003; Saad et al., 2005; Saad, 2006, 2007, 2011a,b; Miller, 2009; Hudders et al., 2014; Kniffin and Shimizu, 2016) as well as, separately, evolutionary studies that focus on questions of managerial importance (e.g., Kniffin and Wilson, 2010; Kniffin et al., 2014, 2015, 2017). Indeed, more broadly, our interests in this article fit with calls for applied evolutionary studies (e.g., Bagozzi and Nataraajan, 2000; Fitzgerald and Danner, 2012; Roberts et al., 2012; Kniffin and Scalise Sugiyama, 2018) since we are interested in the finer-grained question of retail pricing strategies for both buyers and sellers. Independent from our evolutionary framework, a more basic novel contribution of our approach is that we concurrently examine pricing strategies as both a marketing and managerial puzzle with relevance for both customers and employees.

## Pricing Strategies and Customer Reactions

Prior research has examined the degree to which customers and employees differentially value products and the resultant challenge for identifying optimal prices that (i) customers can afford and (ii) allow for a worthwhile profit for the sellers (e.g., Shen et al., 2012). In our case, we are interested in the degree to which people evaluate prices in relation to how much each consumer pays in relation to each other. In effect, we are interested in how people – as customers and employees – respond to pricing systems where there is not a single or uniform price. Our focus on the social dynamics involving frontline employees and customers fits the framework that Grant and Campbell (2007) applied in their studies of service employees and teachers as well as the general call for more attention to managerial problems found in the increasingly large service sector (Anderson, 2006). Our approach to examine (a) how services are delivered (by employees) in relation to customer responses similarly complements (b) Emery and Fredendall (2002) study of automobile garage workers where they found that the organization of workers into teams yielded greater productivity as well as higher customer service ratings.

Prices are understandably a common source of interest for firms whose strategies rely upon satisfied customers (e.g., Grewal et al., 2010); however, the relevance of pricing strategies for employee attitudes has not been closely studied. Even though “there is still much debate” (Edmans, 2012) concerning the relevance of employee satisfaction for firm performance, it seems obvious in retrospect that pricing strategies should be studied in relation to employee attitudes in retail settings, at least, since those frontline employees are *de facto* administrators or brokers of firms’ pricing systems (Sabiote and Román, 2009). While this has not been a focus of prior research, it is noteworthy that in a study of retail grocery stores, Simon et al. (2009) report that employee satisfaction positively relates to (1) sales as well as (2) customer satisfaction with respect to sales. More generally, Simon and DeVaro (2006) find that companies that are recognized as excellent for employees tend to have significantly higher customer satisfaction as well as relatively higher increases in firm value. Similarly, Van Rooy et al. (2011) lay out a “business case” for measuring and attending to employee satisfaction given its importance for positively influencing employee engagement.

In this article, we examine the potential role of pricing strategy as a mechanism in the relationships among customer satisfaction, employee attitudes, and firm performance. While Simon et al. (2009) employ a longitudinal design that permits stronger conclusions about the importance of relationships between employee satisfaction, sales, and customer satisfaction in their study of retail grocery stores, more traditional findings are limited to conclusions that there exist positive correlational relationships among employee and customer satisfaction measures alongside indices of firm performance (e.g., Schneider et al., 1998). Throughout each of these previous studies, researchers have not focused on mechanisms that might account for the positive correlations among firm success and measures of employee and customer satisfaction. In this vein, our research addresses the call that Simon and Gómez (2014) make for “identifying and measuring the specific actions that companies take to increase their level of satisfaction with service, quality, and price.”

## The Current Research

The dearth of previous research concerning variable pricing is sensible since the pricing for most products in the current global retail economy is fixed across customers, notwithstanding publicly advertised coupons or rebates (Tat, 1994). In our case, we present two studies that involve the most prominent retail product where it is still common for customers to pay different prices for precisely identical products. Focusing on the new automobile industry in the United States, we present two studies exploring the question of whether employees prefer fixed or non-fixed pricing. Study 1 is based on a field study of a sample of representative new automobile dealerships while Study 2 focuses more specifically on employee preferences in relation to fixed or variable pricing strategies. Partly because of its nature as a field project, Study 1 trades away precision and power for naturalistic observations whereas Study 2 is designed to be precise albeit outside of a naturalistic environment. Both studies were carried out in accordance with the recommendations of the relevant Institutional Review Board (IRB) – SUNY-Binghamton for Study 1 and Cornell University for Study 2. With IRB approval, informed consent was gained without written signatures for both studies in order to guard participants’ privacy.

## Study 1: Employee Attitudes in Field Study of Pricing-Variable Firms

### Overview

Study 1 examines the degree to which employee attitudes show relationships with pricing strategies within a sample of new automobile dealerships where there is highly visible variation in fixed and flexible pricing systems. Partly due to the relatively unique aspect of new automobile sales in which customers are traditionally expected to negotiate prices with salespeople, Berggren and Nacher (2001) concluded that “the average consumer would rather have a tooth pulled than visit a car dealer” (p. 95). While it is reasonable to expect that the large expenses required for purchasing automobiles can also make such visits unpleasant, there is evidence that pricing dynamics are important as annual Gallup surveys (e.g., Jones, 2005) find that “car salespeople” are held in lowest regard with respect to honesty and ethical standards alongside telemarketers (Strizhakova et al., 2012). In a break with the traditional practice of negotiating each automobile purchase, though, several automobile retailing networks have adopted uniform or “no-haggle” pricing strategies that also promise full transparency to potential customers. Throughout this article, we will interchangeably use “bargaining-free” and “non-negotiated” as synonyms for “no-haggle” pricing since – while each type of phrase carries some implicit value – “no-haggle” does clearly draw upon a negative connotation of haggling as its backdrop. This important variation in pricing strategy warrants closer investigation as a model domain for studying the relevance of pricing in relation to employee attitudes. The variation also offers a real-world opportunity to examine how evolved preferences to avoid being relatively disadvantaged can manifest themselves in contemporary settings.

### Theoretical Background and Research Question (RQ)

Automobiles are often named by manufacturers to communicate competitive and individualist themes (e.g., Nissan’s Maxima, Rogue, and Titan). In contrast with a focus on individuals, some manufacturers have sought to engage customers beyond the transaction of a sale or purchase with the goal of cultivating enthusiastic “fans” (Reeves-Ellington, 1995; Kozinets, 2001). Presumably, firms that pursue this route anticipate that the firm is likely to benefit when customers are likely to form a “brand community” (Muniz and O’Guinn, 2001). For example, if customers become voluntary, unpaid promoters of a given product, then the firm that produces the product will benefit. In fact, companies that can cultivate such a community can enjoy the kind of consumer co-creation described by Johnson et al. (1998) without needing to offer lower prices for early adopters.

Among automobile brands, it is interesting for our focus to consider the case of General Motors’ (GM) ill-fated experimental line named Saturn. Designed partly as a means to break from the manufacturer’s existing labor and franchise agreements with its first cars being sold in 1991 (Rubenstein and Kochan, 2001), Saturn did have important successes even though it ultimately folded in bankruptcy in 2009. While there is debate with respect to whether GM was fully and consistently committed to the success of Saturn (e.g., Ingrassia, 2009), the relevant patterns for our interest are that (1) Saturn was uniquely committed for most of its existence to fixed or no-haggle pricing for customers and (2) Saturn excelled in customer satisfaction. For example, Saturn consistently outranked expensive, luxury brands in customer satisfaction for “sales experience” and earned the highest score among products distributed by GM, Ford, and Chrysler in an industry-wide survey of customer satisfaction conducted near the time of its bankruptcy (e.g., JDPA, 2002). As a tangible measure of the brand’s reputation, Rhee and Haunschild (2006, p. 109) report that Saturn products consistently had better depreciation rates than other brands produced by manufacturers headquartered in the US. In the context of celebrating the “dedication-based” bonds that Saturn sought to cultivate with its customers, Bendapudi and Berry (1997) describe the “homecoming” events that the firm hosted where thousands of Saturn owners traveled to the original factory in Spring Hill, Tennessee to engage with the brand and with each other.

As the first major line of automobiles in the US to be sold according to uniform pricing rules, Saturn’s practice of “no-haggle” pricing offered a chance to connect with customers without the negotiations that characterize most automobile dealerships. As Mills et al. (2001, p. 130) interpreted, haggling would have been disruptive to the community that the company clearly sought to build. Likewise, one of Saturn’s earlier Presidents was clear to oppose discounts and rebates on the grounds that the practice would “hurt the people who now own our cars by lowering the value of their cars” (Truett and Teahen, 2002). Stated in the affirmative, the customer satisfaction ratings suggest that the lack of haggling and the uniformity of the prices actively facilitated a feeling of community with the company and with “fellow” Saturn owners with whom buyers could confidently assume relatively shared buying experiences. This interpretation fits with previous research showing the potential for price discounting to invite customer perceptions of low product quality (e.g., Srivastava and Lurie, 2004; Darke and Chung, 2005) and negative feelings (e.g., guilt) in relation to customers who do not pay discounted prices (Gelbrich, 2011). In an evolutionary view, given the important effects known to occur because of consumers comparing themselves to one another (e.g., Winterich and Nenkov, 2015), Saturn’s pricing was designed to avoid anyone feeling like they lost in relation to other customers.

Independent from Saturn’s experiences, it is noteworthy – and unsurprising in light of their success with respect to customer satisfaction – that the practice of fixed or no-haggle pricing has been adopted by other firms. Most notably, Toyota’s Scion brand is marketed to customers as having a “pure price” that is not subject to haggling or negotiations. Similarly, CarMax – a successful retailer that has grown to more than 100 dealerships across the US – highlights their “no-haggle” pricing in all of their promotional materials. In fact, CarMax’s trademarked slogan focuses on this aspect of their sales process: “The way car buying should be.” Indeed, Tesla – as an upstart among auto manufacturers that has retained control over its sales network with apparent success, to date – follows this same non-negotiated pricing approach (e.g., Bhattarai, 2016).

Against the backdrop of evidence that customers appear to respond favorably when firms seek to engage them cooperatively through the form of fixed or uniform pricing – in a market, at least, where the tradition involves bargaining or negotiating over prices, previous research has not considered the possibility that firms that practice fixed pricing might also tend to have employees who are more cooperative or prosocial. Building on earlier studies that have juxtaposed employee attitudes alongside customer satisfaction (Simon and DeVaro, 2006; Simon et al., 2009) and found important crossover effects (Hui et al., 2007; Zimmerman et al., 2011; Kao et al., 2014), we examine whether cooperative employee attitudes will be more likely to be found within firms that offer fixed pricing. Our question presumes that since fixed pricing is relatively community-oriented, then we anticipate that the employees who administer such a pricing system will also tend to demonstrate more community-oriented or prosocial attitudes.

**RQ1.** Do firms that implement uniform pricing for customers tend to have employees who are more prosocial than firms whose pricing is based on individualized negotiations?

### Methodology of Study 1

#### Research Design

A sample of three new automobile dealerships in a shared market in the Northeastern United States granted us access for on-site research intended to improve their operations. Dealership A was affiliated with a single domestic manufacturer, Dealership B was the franchise for two domestic companies and one imported brand, and Dealership C served three domestic firms and one imported line. With the benefit of results from proprietary manufacturer-sponsored customer surveys, we know that each of the dealerships were representative of their affiliated manufacturers’ dealerships in terms of customer satisfaction. We also know that Dealership A – the one of the three that practiced fixed pricing – had superior customer satisfaction ratings when compared with Dealerships B and C.

In advance of developing and finalizing a quantitative survey for administration to the dealerships’ managers and employees, one of us spent more than 12 h a week for eight consecutive weeks interviewing and observing employees and customers at the three locations. In addition to gaining insights into the dealerships’ operations, our presence at the dealerships also helped engage employees to participate in the questionnaire study that we conducted. Among the dimensions that we were able to study through on-site qualitative research as advocated by Cohen (1999), the pricing systems as well as employee dispositions emerged as salient features that warranted closer quantitative study. **Appendix A** provides the full set of items that we assessed through the questionnaire and the specific measures that we analyze in this article are described in the next section (“Measurement of Variables”). Similarly, **Appendix B** provides descriptive statistics for each of the items in **Appendix A** as well as the analytic outputs that inform our Results.

#### Participants

Seventy-four of 117 employees and managers working at Dealerships A, B, and C returned questionnaires, with response rates of 88% (*n* = 19), 54% (*n* = 26), and 57% (*n* = 29), respectively. The average age of respondents across dealerships was 39 years and the majority were men (79%). The average tenure for their current jobs was 5.6 years. There were no significant differences with regard to age and tenure across dealerships – or across departments. The range of respondents across departments and dealerships appears representative based on comparisons with direct observations.

### Measurement Variables

#### Social Value Orientation (SVO)

As an indirect assessment of whether people are prosocial, individualistic, or competitive (or none of the above), the SVO scale (Van Lange et al., 1997) is based upon a series of nine hypothetical situations wherein participants are asked to differentially allocate a set of resources between themselves and another individual. Participants were asked to complete the SVO two separate times – first in relation “to the dealership” and second in relation to a randomly paired (and hypothetical) “customer of your dealership.” Each set of the 9-scenario prompts are found in **Appendix A** as Questions 19 and 20, respectively.

#### Primary Psychopathy

In order to measure more general dispositions of dealership employees, we chose the Primary Psychopathy scale given its recognition as a measure of antisocial intention (Levenson et al., 1995). For this measurement, respondents are presented with a set of 16 statements and are asked to state their level of agreement or disagreement. Primarily, the questions test the extent to which individuals place their own interests above the interests of others and use a 5-point Likert scale in which 1 represents agreement and 5 measures disagreement. The items are found in **Appendix A** as Questions 21–36.

#### Attitudes and Values

We employed an additional measure of Attitudes and Values based on earlier research conducted by Reeves-Ellington (1996, 1998) that was aimed at viewing differences among individuals with regard to their views of human nature (trustful-distrustful), their relation to the external environment (dependent-interdependent), their approach to human relations (structured-unstructured), and time (present-future). Utilizing the same 5-point scale described in the previous section, the full set of items are found in **Appendix A** as Questions 4–14.

#### Inclusion of Other in Self

As a measure of the degree to which people feel they are “part of” something or someone else, we modified the Inclusion of Other in Self (IOS) scale (Aron et al., 1992) in order to assess respondents’ self-identification with (a) the dealership where they worked, (b) the average customer, and (c) employees who work at other new automobile dealerships. The three items are found in **Appendix A** as Questions 1–3.

#### Teamwork Perceptions

To complement the IOS measures, we asked a set of four direct questions (in **Appendix A** as Questions 15–19) to assess the degree to which respondents felt there was “a spirit of teamwork” in, separately, the department or dealership where they work; and, a complementary pair of questions assessing whether they “feel like an important member” of their department or dealership.

### Results of Study 1

#### Social Value Orientation (SVO)

**FIGURE 1:**
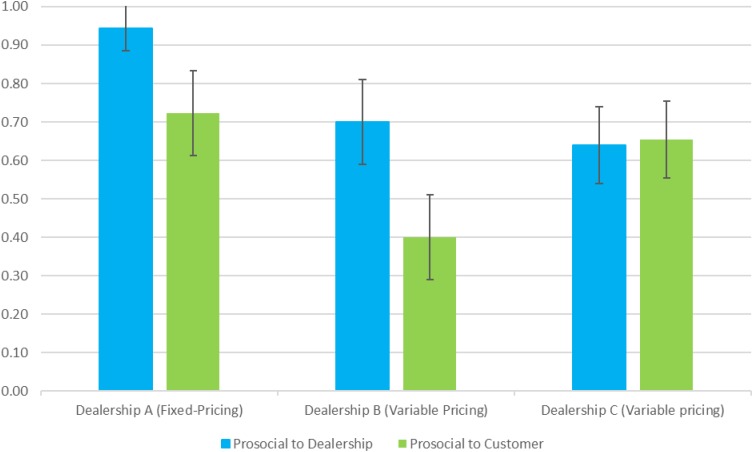
Percentage of Prosocial Employees Across Dealerships as Measured by Social Value Orientation (SVO). Error bars represent the Standard Error (SE) for each value. The upper limit of the SE for the left-most bar is 1.0.

As illustrated in **Figure 1**, data from the SVO ratings, which permit identification of employees as being prosocial (or not) in relation to, separately, the dealership as a whole and to the dealership’s customers, showed patterns that appeared to answer RQ1 affirmatively; however, the patterns were not significantly different among the dealerships. Specifically, the distribution of employees categorized as prosocial versus either individualistic or competitive at the between-dealership level generated *F* = 2.839, *p* = 0.066 in relation to the dealership and *F* = 2.436, *p* = 0.096 in relation to the customer.

Focusing exclusively on employee prosociality in relation “to the dealership,” it is notable that a majority of respondents at each location were found to be prosocial to their firm, but the percentage at Dealership A was considerably higher (94%) compared to the ratio at the other two dealerships (70 and 64%). When Dealerships B and C were collectively compared with Dealership A on this trait via a *post hoc* contrast on the main ANOVA, the difference between the two groups is significant (*t* = 2.30, *p* = 0.025).

Focusing on SVO in relation “to the customer,” there was not a significant difference (*F* = 1.706, *p* = 0.196) when Dealership A is compared directly against the aggregate of Dealerships B and C. It is interesting to note that the percentage of prosocial employees at Dealership B – in relation to the customer – is the lowest SVO value in **Figure 1**.

The limited tendency for employees at Dealership A to appear more prosocial through the SVO measurement is consistent with the fact that employees at Dealership A participated at a higher rate, nominally at least (*X*^2^ = 3.01, *p* = 0.082), when compared with response rates at each of the other two dealerships. Prior research utilizing the “lost-letter” methodology (e.g., Wilson et al., 2009) suggests that the response rates that we observed across the three dealerships possibly reflects a stronger prosocial orientation among the employees and managers at Dealership A or a tendency for the least prosocial employees from dealerships B and C to not participate; however, we do not have data to test these speculations.

#### Primary Psychopathy

For the full Primary Psychopathy scale (Cronbach’s alpha = 0.86), there was not a significant difference across dealerships (*F* = 2.77, *p* = 0.07); however, similar to the findings for SVO in relation “to the dealership,” the *post hoc* contrast between Dealership A and the aggregate of Dealerships B and C is significant (*t* = 2.21, *p* = 0.030).

As a follow-up to the comparisons involving Primary Psychopathy scores, we compared responses to each of the sixteen questions and found there were differences (as reported in **Table 1**) for three out of sixteen of them, using unadjusted significance levels, with a pattern whereby workers at Dealership A disagreed more strenuously than employees at other dealerships with the following statements: (1) “In today’s world, I feel justified in doing anything I can get away with to succeed” (*F* = 3.725, *p* = 0.029); (2) “My main purpose in life is getting as many goodies as I can” (*F* = 3.180, *p* = 0.048); and, (3) “People who are stupid enough to get ripped off usually deserve it” (*F* = 8.279, *p* = 0.001). When the significance thresholds are adjusted according to Holm (1979) to reduce the risk of false positives given the 16 question-specific calculations, only question (3) remains significant whereas both (1) and (2) are above the significance threshold. More specifically, using the Holm adjustment, the 16 item-specific *p*-values are ordered from lowest to highest and then divided by the inverse-ranking such that (for example) a pre-determined significance value of 0.05 is translated to 0.003 (or 0.05 divided 16) for the item (among the set of 16) with the lowest *p*-value. Consequently, since there is a pattern suggested only by (3), it is appropriately recognized as very faint.

**Table 1:** Descriptive statistics for Decomposed Items Measured in Study One^1^.

	Dealership A (fixed-pricing)	Dealership B (variable pricing)	Dealership C (variable pricing)
**Significantly different primary psychopathy items (3 of 16)** (1 = Agreement; 5 = Disagreement)	***M (SD)***	***M (SD)***	***M (SD)***
In today’s world, I feel justified in doing anything I can get away with to succeed	4.61 (0.50)	4.08 (0.88)	4.10 (0.62)
My main purpose in life is getting as many goodies as I can	4.50 (0.62)	4.29 (0.75)	3.97 (0.78)
People who are stupid enough to get ripped off usually deserve it	4.67 (0.59)	4.25 (0.79)	3.72 (0.88)
**Significantly different attitudes and values items (4 of 11)** (1 = Agreement; 5 = Disagreement)	***M (SD)***	***M (SD)***	***M (SD)***
When customers get treated badly by store salespeople, they should expect it; after all, we are all human	4.95 (0.23)	4.32 (0.90)	4.38 (0.90)
You must think of yourself if you want to get ahead	4.05 (0.97)	3.25 (1.23)	3.28 (1.00)
It is OK to treat groups of people from other countries or cultures differently than we do people like ourselves	4.74 (0.56)	4.08 (0.78)	4.28 (0.75)
Today, most companies cannot compete without special treatment	3.74 (0.87)	3.79 (0.88)	3.17 (0.76)

*^1^Descriptive statistics for each of the 16 Primary Psychopathy items as well as each of the 11 Attitudes and Values items are reported in **Appendix B**.*

#### Attitudes and Values

For the 11-item survey of Attitudes and Values, we did not use the factors (trustful-distrustful; dependent-interdependent; structured-unstructured; and, present-future) previously applied by Reeves-Ellington (1996, 1998) because the reliability measure (i.e., Cronbach’s alpha) for each factor did not reach significance. Beyond noting that Reeves-Ellington’s prior use of the questions was not validated as a standalone scale, we can point out that previous research employing the questions enjoyed the power of substantially larger samples. Instead, as with our analysis of Primary Psychopathy, we decomposed the full set of questions and analyzed responses to each item across dealerships. Before adjusting for the multiple comparisons of 11 items within this subset of questions, the four statements that drew significantly different responses across dealerships were (as reported in **Table 1**): “When customers get treated badly by store salespeople, they should expect it; after all, we are all human” [*F*(2,70) = 4.075, *p* = 0.021]; “You must think of yourself first if you want to get ahead in life” [*F*(2,69) = 3.790, *p* = 0.027]; “It is OK to treat groups of people from other countries or cultures differently than we do people like ourselves” [*F*(2,69) = 4.582, *p* = 0.014]; and, “Today, most companies in my country cannot compete without special treatment” [*F*(2,69) = 4.451, *p* = 0.015). As **Table 1** specifies, for three of the four questions, employees at Dealership A reported greater disagreement than workers at the other dealerships. For the fourth question, employees at Dealerships A and B differed significantly from personnel at Dealership C. When the significance thresholds are adjusted for these comparisons according to Holm (1979), none of them remain significant.

In contrast with the faint and preliminary tendencies that appear in **Table 1**, the measures that we collected for IOS and Teamwork Perceptions did not show significant variation among the dealerships. In **Appendix B**, we report the raw output for these comparisons, which arguably affirm the faintness of the inferences to be drawn from the mix of **Figure 1** and **Table 1**. More specifically, we can report that there was non-significant variation among the dealerships when we conducted ANOVAs for IOS-dealership (*F* = 0.01, *p* = 0.99), IOS-customer (*F* = 0.44, *p* = 0.64), IOS-employees (*F* = 0.89, *p* = 0.42) and for perceived teamwork at the department (*F* = 0.71, *p* = 0.50) and the dealership (*F* = 0.31, *p* = 0.74) where one works and for feeling like an important member of the department (*F* = 0.38, *p* = 0.69) and the dealership (*F* = 0.11, *p* = 0.90) where one works. On the other hand, it is also arguable that – in relation to RQ1 – the measures reported in **Table 1** are more directly relevant to the question of employees’ prosociality than the IOS and Teamwork Perception measures since the latter set of questions relate mainly to personal identity.

Taking stock of the findings that we report from the field study that we conducted for Study 1, the patterns highlighted in **Figure 1** for SVO along with the items reported in **Table 1** that are related to Primary Psychopathy as well as Attitudes and Values offer faintly positive and preliminary support for the view that employees at Dealership A are more prosocial than employees at Dealerships B and C. While Study 1 is not designed to test for any causal relationships involving Dealership A’s workforce partly because there are numerous other dimensions of difference across the sample of dealerships (e.g., they vary in size and types of vehicles they sell), the findings – based on naturalistic field data – provide very faint but suggestive correlational evidence concerning employee attitudes and firm-level pricing strategies.

## Study 2: Attractiveness of Price Discipline for Employees

### Overview

While a significant aspect of Dealership A’s strategy in Study 1 involved fixed pricing for customers through which no buyers felt relatively disadvantaged, we conducted Study 2 to measure attitudes among prospective employees for automobile dealerships that do and do not negotiate prices with customers. While the field study permits a preliminary test of RQ1 with the benefit of naturalistic observations, Study 2 permits us to focus very specifically on employee preferences in relation to pricing strategies. Study 2 also allows us to undertake a hypothesis-testing approach to follow on the exploratory and discovery-focused nature of our Study 1 analyses (see Bamberger, 2018 for more on this distinction). Additionally, our juxtaposition of a field study with the artificial approach that we adopted for Study 2 offers a parallel to the recent review by Starmans et al. (2017) wherein they report that responses to inequity in large-scale field settings tend to be very different (i.e., muted) when compared with responses to disparate treatment in artificial experimental settings. While the scope of the small-scale dealership-level Study 1 is many levels of organization lower than the national-level data on which Starmans’ et al. derive much of their contrast, there is nevertheless definite value gained by complementary methodological approaches. For example, while national-level survey analyses tends to presume that any patterns reveal underlying preferences, it is always plausible – perhaps especially with respect to questions of inequity – that such patterns also partly reflect processes that are not preference-revealing; consequently, lab studies through which individual participants are able to directly indicate their preferences without the noise and history of broader social environments are valuable complements.

### Theoretical Background and Hypothesis 1

In the context of research we presented in the introductory section, it is worthwhile to recognize that fixed or bargaining-free pricing removes the possibility of comparison and competition among customers for a given product. Since automobile purchasing is among the few domains where negotiating is not uncommon and customers can get “a good deal” (relative to other customers) on identical products purchased by other customers, it is interesting and worthwhile to explore further. We can start understanding why an evolutionary perspective anticipates concern with one’s relative standing – on the sale or purchase price of an automobile – by drawing out the underlying analogy between measures of cultural success and measures of genetic fitness.

Most generally, evolutionary anthropologists and psychologists have found positive relationships between a person’s “cultural success” and their reproductive fitness (e.g., Irons, 1979). Indeed, more recently, in a meta-analysis drawing upon patterns found in 33 non-industrial societies, von Rueden and Jaeggi (2016) show a robust positive relationship between status and reproductive success. In slight contrast, Stulp et al. (2016) are clear to highlight a variety of reasons why such positive relationships warrant closer investigation (e.g., there are substantial differences among ethnic groups); however, they also find a general positive relationship – in a contemporary industrial population – between wealth and fertility for men and women.

At an ultimate level, it is sensible that people who “succeed” in a given society – whether it is pre- or post-industrial – will tend to have their success reflected genetically, although both the invention of contraception (Perusse, 1993) and the contemporary tendency for parents with relatively high levels of wealth to invest relatively heavily in a small number of offspring (Kaplan, 1996) undoubtedly affects the relationship. At a more proximate scale, it makes sense that the dimensions on which people succeed will vary significantly across context. For example, success among foragers or horticulturalists will certainly be measured differently than success among people living in a contemporary urban environment. It is against this backdrop that we can recognize that – independent of the specific currency being sought in a given environment – there is consistent evidence of generally positive ultimate outcomes as measured by reproductive fitness (von Rueden and Jaeggi, 2016).

Given the recognition that getting “a good deal” is a private good that exists in markets where consumers can haggle or negotiate for a price that is better than others pay, it is clear that (a) people would tend to enjoy getting a good deal all else equal and that (b) no one is at risk of becoming a loser relative to other buyers – or sellers – when prices are fixed. While previous research has focused on consumer attitudes to differential pricing strategies (e.g., Frank, 2001), we examine the question of whether employees also will tend to demonstrate a preference for retail workplaces where prices are not subject to negotiating. Given the conventional view of salespeople as predatory, our interest complements Grant and Campbell (2007) finding that service employees appear to have better attitudes toward their work when they believe they are helping rather than harming others. Our research question also builds upon Colarelli and Dettmann (2003) identification of preferences to avoid loss as a part of our evolved heritage.

We can highlight further that our focus – given its contemporary relevance – on employees, rather than people in more generic environments, is important for our approach since (a) most retail workplaces do offer people a chance to work in sales roles that offer no reward for negotiating while (b) there are some examples, most notably automobile dealerships, where people in sales roles are traditionally expected to engage in variable or negotiated pricing. We do not expect that it would be adaptive – or reasonable to hypothesize – that “owners tend to prefer operating in environments where prices are uniform across customers” since owners are vested in earning profits. In contrast, employees can reasonably be expected to adapt to the lower stakes that are typically involved in their positions – as front-line employees who interact with customers – to prefer more equitable pricing that is devoid of bargaining. While it is reasonable to recognize that interactions that are free of bargaining are less expensive for employees (e.g., in terms of cognitive load) and therefore predicted on simple grounds of saving energy, we designed Study 2 to focus exclusively on preferences to establish a baseline upon which future research could build.

**H1.** Employees tend to prefer working in an environment where customers are uniformly charged as compared with environments where each customer pays a different price based upon negotiations.

### Methodology of Study 2

117 adults (43 women and 74 men with an average age of 26 years old [*SD* = 11.4]) were recruited from an online population validated for research purposes (Buhrmester et al., 2011) to participate in Study 2 in exchange for monetary compensation.

We instructed participants to: “Please assume that your best friend is most interested to work in a retail sales position after graduating from college. On a scale of 1 to 7, please indicate the degree to which your friend would like to work for either of the companies specified below (1 = no interest at all, 4 = moderate interest, 7 = very interested). Please be sure to assume that your best friend’s income would be the same at each of the organizations and that both organizations have the same financial health and prospects for the future.” For the instructions, we asked participants to describe their best friend to avoid potential response bias and we specified that income and employer stability would be constant to help control for potentially extraneous assumptions.

For the two companies, participants were asked to rate the level of interest in a “Retail organization where employees are paid fair wages and customers are charged uniform prices” and a “Retail organization where employees are paid fair wages and customers are charged variable pricing that depends upon the outcome of their individual negotiations with salespeople.”

After participants completed the ratings, we asked them an open-ended question to indicate the rationale for any preference they reported.

### Results of Study 2

**FIGURE 2:**
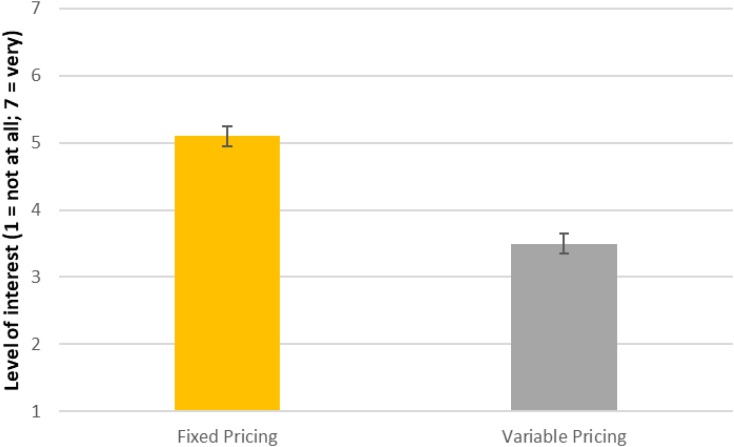
Results for Study Two indicate higher “Best Friend’s Preference for Working at an Organization” with fixed pricing (Means and Standard Errors reflected in Figure).

As illustrated in **Figure 2**, participants reported a significantly greater preference for working in the environment where prices were fixed rather than negotiated (*t* = 7.20, *p* < 0.001). To help test for the robustness of this effect, we examined whether sex and age differences might drive the overall finding and the pattern reported in **Figure 2** (a) appears significant for, separately, both women and men and (b) does not appear to be significantly influenced by age. We can infer from these patterns that people expect that employees will also tend to gain greater enjoyment from working in an environment where prices are not negotiated and where it is assumed there are no benefits associated with negotiating. We can add a more basic frequency count to note that 22 respondents rated the “variable pricing” retail organization as more attractive; 23 rated them equally; and, 72 rated the no-haggle pricing organization as more attractive, given a situation where there was no reward for employees to engage in bargaining.

In response to our open-ended question, while we did not transform the qualitative data into quantitative forms to support systematic analyses, we do provide a full listing of all responses alongside each respondents’ preferences in **Appendix B** and we can observe that responses often fit with H1’s prediction that people will prefer working in the fixed-price dealership. Examples include “It would be less stressful if customers all receive the same price,” “My friend would not like that some customers would end up paying more or less than others based on who they happen to talk to,” and “I feel it would be more easier [*sic*] and harmonic to work in an environment with set prices.” Further, a preference for fairness was demonstrated by people who indicated that “Uniform prices are the most fair,” “Everyone appreciates fairness,” and “Customers should pay equal prices for equal services/goods.” One participant interestingly went so far as to anticipate potential legal problems that can exist when prices are flexibly negotiated: “If the customers are charged a uniform price, it’s easier to keep track of how many items or units were sold. I think the customers would be treated fairly no matter their status, race, gender or sexual orientation. A uniform price seems direct and transparent.”

Notably, while the open-ended responses offer largely qualitative data to consider, it is interesting in relation to the perspective that energetic-savings should predict that responses to our vignette lean strongly to no bargaining, participants did not predominantly account for their preferences for fixed pricing as a response to the greater transaction costs that are entailed by negotiating. There is, though, suggestive evidence that people who indicated preferences for fixed pricing were at least partly motivated by community-oriented concerns that often highlighted the value of fairness (e.g., 11 people who favored uniformed-pricing – or approximately 10% of the full sample – used the root-word “fair” in their responses).

## Discussion

Via two complementary studies, we have been able to consider the relevance of pricing strategies in relation to employee attitudes. In Study 1, while the results are only faintly suggestive of a positive relationship, we examine the question of whether employees at a dealership with high customer satisfaction and fixed pricing are more prosocial than comparable employees at other dealerships. Through Study 2, we focus on preferences held by potential employees for positions at a retail location and we find that people tend to prefer working in the environment in which prices are fixed, if, at least, they are assured that bargaining on price will not yield any direct benefits. Our focus on employee attitudes and preferences in relation to pricing strategies and, indirectly, customer satisfaction offers a novel contribution to identifying a specific mechanism – fixed or non-negotiated pricing – through which firms attempt to align with high employee and customer satisfaction (Simon and Gómez, 2014).

The findings that we present add value to prior studies that have established a general positive relationship between the sense of fairness that employees feel within an organization and their tendency to engage in organizational citizenship behaviors (e.g., Messer and White, 2006). First, our studies illustrate the importance of considering interrelationships among employee, customer, and organizational interests – a set of connections that have increasing relevance as the service sector of our economy continues to grow (e.g., Anderson, 2006). Second, our studies substantiate the utility of anticipating and understanding evolved preferences in relation to contemporary business problems. As a complement to others who have focused on the importance of one’s standing relative to one’s peers in terms of income or consumption (e.g., Frank, 2001, 2012; Luttmer, 2005; Saad, 2007, 2011a,b), our studies suggest that people – as frontline or contact employees – tend to prefer situations where they would not need to enact disparities among customers with whom they develop a bond during the sales process. In other words, while customers do not typically form a cohort amongst themselves, the relationships that are formed between customers and retail staff appear to be sufficiently important that employees appear more likely to prefer that customers “win” through a pricing system that does not create losers.

To compare our studies in closer detail with recent research on employee responses to pay dispersion within the firms that employ them, it is notable that Shaw (2015) finds that high-performing individuals tend to quit more frequently when their employers pay relatively “compressed” or similar wages (i.e., not rewarding high performers) while low-performing individuals tend to quit more frequently when their employers pay employees closer to their marginal product (i.e., pay for performance). The pattern that Shaw reports is arguably consistent with Frank (1984) observations that lower-performers need to be paid more than their marginal product in order to accept their lower status in the workplace; however, Shaw’s findings suggest that – at the organizational level – it might be beneficial if lower-performers vacate their positions. In the case of the salesperson-customers relationships that we examined in this work, it is imaginable that customers who are accustomed to “working hard” for a low price (e.g., by researching and/or by committing time to haggling and/or comparison-shopping) will feel they have earned the right to a lower price and, in that case, walk away (or not enter) a dealership known for uniform pricing. Of course, the frequency of interaction among most co-workers is different in kind from the isolated and relatively rare interactions that occur between any specific salesperson-customer pairings; however, the juxtapositions as they relate to questions of fairness, equality or uniformity, and interpersonal relationships are provocative and intriguing.

### Limitations and Future Directions

We conducted two studies as a means of addressing limitations with each of them. Study 1 is based on naturalistic field data; however, the noise of Study 1 is that there are inevitably additional variables of difference across the three dealerships that our analysis does not incorporate and, further, the study has low statistical power since dealerships are relatively small-scale organizations. Among other unmeasured variables of potential importance, employee salary levels as well as hours-worked-per-week are illustrative measures that our analysis of Study 1 is not able to consider. Study 2 avoids the noise of a field study and focuses on a precise albeit hypothetical situation.

Together, the two studies provide an interesting parallel to Starmans et al. (2017) recent work highlighting divergent responses to inequity in field versus lab settings since our interpretation of our findings is that (a) there exist faint but extant suggestions of prosociality in the field setting of Dealership A alongside (b) a relatively stronger pattern in favor of fixed or uniform pricing in the context of Study 2’s hypothetical choice. Our assessment of these relationships is that the focus of Starmans et al. (2017) on highly aggregated patterns (e.g., national-level attitudes to inequity) misses the finer-grained type of organizational or firm-level field setting that we examined in Study 2. Indeed, such an assessment fits with prior work (Frank, 1984; Luttmer, 2005; Kniffin, 2009; Norton, 2013) that has highlighted that proximity or familiarity to co-workers or teammates can be key to how someone responds to unequal allocations of value. In this respect, we hope that our work draws attention to a middle ground in the divergence that Starmans et al. (2017) highlight between laboratory studies and national-level field-based comparisons.

Additional limitations to identify in each of our studies include the fact that fixed pricing systems do not necessarily mean that customers are getting an absolutely good deal since the fixed prices could consistently be generating higher profit margins for the seller. This explains why we regularly highlight that the perceived “good deal” that people appear to perceive through fixed pricing in the dealership context is focused on “good” relative among the buyers. It is also true that firms with fixed prices for a basic type of product might have a variety of “extra” or “add-on” products and services through which they generate highly variable prices across customers who, in the case of an automobile dealership, all drive away from the dealership in the same type of vehicle. More specific limitations include Study 1’s non-random ordering of questions such as the two SVO questions (first “to the dealer” and second “to the customer”) since random ordering would have been ideal and could have produced different results.

In Study 2, we also acknowledge the possibility that “fair” appeared in a relatively high percentage of the rationales that participants provided since “fair wages” was part of the scenario that they were rating. Indeed, just as ordering questions randomly would have been ideal for Study 1, it is plausible that Study 2 also produced some degree of responses that were biased artifacts of the stimulus. For example, alternative explanations for the findings in Study 2 include the fact that there is less transaction cost (and perhaps less cognitive load) when sellers do not need to negotiate with potential buyers; however, as we note above, the relative absence of such an explanation in the stated rationales invites curiosity on the degree to which an awareness of energy or transaction costs was important for Study 2’s findings.

Among avenues for future research that are highlighted by our studies, it seems likely that there are individual differences – perhaps correlated with personality differences such as Machiavellianism (Wilson et al., 1998) – with respect to how much an employee might prefer to administer a fixed pricing system. On a broader scale, our focus on participants in the US raises the possibility that the same patterns would not be found across cultural groups. The evolutionary argument underlying H1 proposes that there exists a universal preference among employees to avoid outcomes that leave anyone subordinate; however, given that the US is typically classified as “individualist,” future research conducted in more “collectivist” societies (e.g., Lykes and Kemmelmeier, 2014) would be able to test whether this pattern exists within populations outside of the US. Even within the US, we expect that there exists dimensions of individual difference along which subpopulations of people seek the relative risk and reward of negotiated pricing. Indeed, while our Study 2 was not designed to explore the potential existence of sex differences in orientation to variable or non-negotiated pricing systems, future research should certainly explore the degree to which men might prefer variable pricing more than women given that men tend to demonstrate more variable fitness outcomes than women.

Finer-grained analyses that are recommended by our studies include consideration of additional mechanisms that might account for the preferences that we report in Study 2. For example, given previous research showing that the trust of salespeople is important for sales (Kennedy et al., 2001), we can speculate that salespeople’s preferences to be liked and trusted by customers explains at least part of the patterns that we report in this article. It is also plausible that frontline employees prefer fixed pricing because it protects them against pressure by ill-intended supervisors who might otherwise feel free to encourage predatory pricing (e.g., Dubinsky et al., 2004) that do yield relative losers and winners among the customers. Similarly, while RQ1 focused on generic prosociality being important in relation to employees working in an organization with non-negotiated as compared with variable pricing, future research should examine the degree to which self-identification or prosociality specifically in relation to the firm might be more important.

Outside of the traditional retail environment, it is valuable to consider our findings in relation to domains such as education where it is plausible that teachers who provide the same grade to all students will tend to have more job satisfaction than teachers who actively and closely discriminate student performance through grading (Tierney, 2013) provided that there is a basic level of skill and effort demonstrated by all of the students. To extend the analogy with our studies, teachers (as salespeople) would be extending the same product (grade) to customers (or students) whose variable ability and effort would normally be valued differentially and worth the equivalent of a wide range of new and used vehicles. It is imaginable that mid-level managers might also have greater job satisfaction in workplaces where they rate all of the subordinates as equivalent; however, we would expect that market forces are stronger in for-profit ventures and such managers’ resistance to grading employees would be unsustainable. Similarly, within academia, we expect that department chairs – in universities where performance-based pay increases are traditional – might enjoy a degree of private or individual satisfaction if they were to rate (and reward) all faculty uniformly, but that such satisfaction would be fleeting in the face of concerns over justice (Kimes and Wirtz, 2003; Wirtz and Kimes, 2007; Schlereth et al., 2011) and likely counterproductive over time (Shaw, 2015). Each of these analogous situations are examples where “everyone can win” in a given round or two of deliberations, but problems will tend to emerge when the relationships are more serial – thus pointing to a limitation for drawing generalizations about dealership sales wherein sales tend to be far from frequent (i.e., salespeople do not typically interact with the same set of customers each year in the same way that employers and employees might engage in periodic reviews one or more times each year).

## Conclusion

New automobile dealerships provide an ideal domain for studying the role of pricing strategies in relation to employee and customer satisfaction partly because of the variation that exists with respect to pricing as well as the relatively high stakes that are involved in the transactions. While there are relatively few other markets where it is common to negotiate prices in the US, the findings that we report in this article help to account for the dominance of fixed pricing for most consumer-focused firms. In the market of consumer electronics, for example, where customers do not regularly negotiate prices but there is often steep pricing competition, it is interesting in light of our studies that Apple has cultivated a community of fans while concurrently maintaining relatively strict price discipline among the firm’s retail partners. Our studies suggest that these practices cultivate community-oriented attitudes and generally higher preferences among a firm’s employees – in retail settings, at least – in addition to its customers.

While previous applications of evolutionary frameworks to contemporary business problems tend to focus on consumption – with a particular focus on the relevance of sex differences (e.g., Saad, 2004; Kruger and Byker, 2009; Miller, 2009) – we build on that work to consider how customer and employee preferences in relation to pricing systems can be better understood. In the case of fixed pricing, it is clear that the system offers an opportunity for all buyers and sellers to feel like winners – or at least avoid the potential of feeling like a loser – in relation to each other. Beyond providing a basis for understanding how pricing systems can be affected by evolved preferences (e.g., to avoid the risk of being relatively disadvantaged), our analysis also highlights the value of considering the effects of consumer-oriented practices such as pricing on the experiences of front-line employees whose job involves regular, daily customer interactions.

## Author Contributions

KK conducted the research and wrote the article. RR-E and DW supervised the research and contributed to the article.

## Conflict of Interest Statement

The authors declare that the research was conducted in the absence of any commercial or financial relationships that could be construed as a potential conflict of interest.
