# Supplementary material for: When Everyone Wins? Exploring Employee and Customer Preferences for No-Haggle Pricing
Source: Front Psychol. 2018 Sep 6;9:1555. doi: 10.3389/fpsyg.2018.01555 (PMC6136271; doi:10.3389/fpsyg.2018.01555)
Supplement: Supplementary file 1 [file Data_Sheet_1.pdf]

Appendix A.

1. Please circle the picture below which best describes your relationship with the **dealership** where you work:

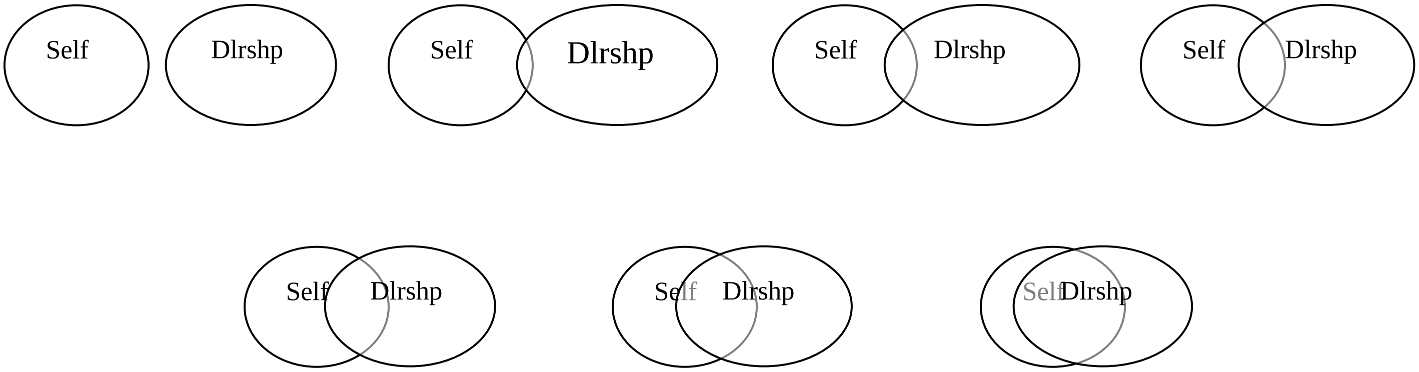

2. Please circle the picture below which best describes your relationship with the **average customer** that you serve:

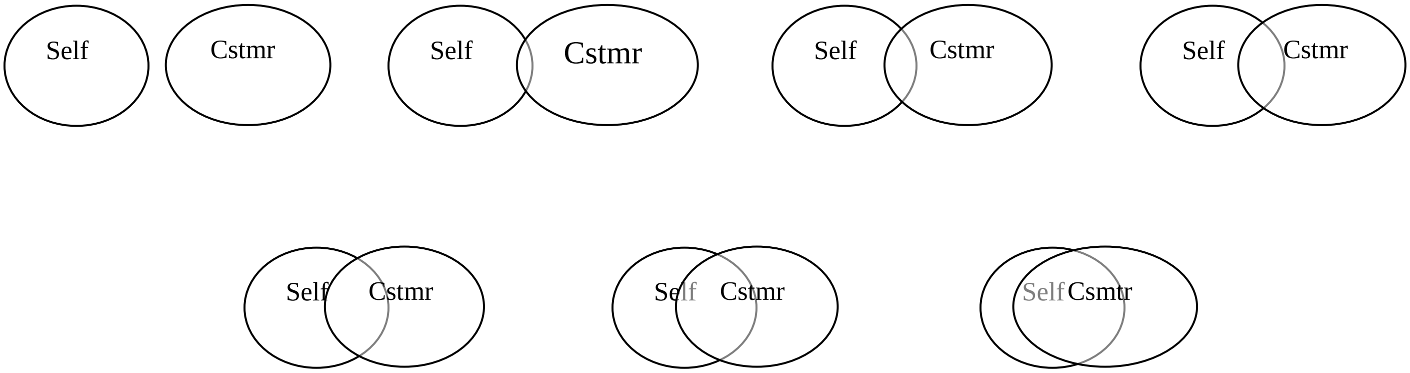

3. Please circle the picture below which best describes your relationship with **employees of dealerships in the region** (not including those owned by the owner of the dealership where you work):

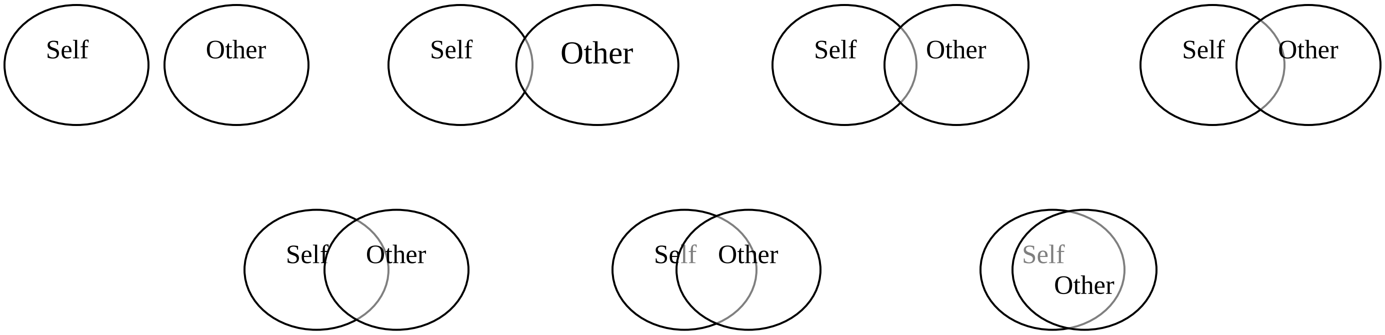

Please state your opinion on the following questions by circling a single number 1 through 5, depending upon the extent to which you agree or disagree with each statement.

4. When customers get treated badly by store salespeople, they should expect it; after all, we are all human.

Strongly agree	Agree	undecided	Disagree	Disagree strongly
1	2	3	4	5

5. Most people can rarely be trusted.
1 2 3 4 5

6. Everyone cheats on their taxes, at least a little bit.
1 2 3 4 5

7. You must think of yourself first if you want to get ahead in life.
1 2 3 4 5

8. Successes of other people are a threat to one's power in an organization.
1 2 3 4 5

9. It is OK to treat groups of people from other countries or cultures differently than we do people like ourselves.
1 2 3 4 5

10. There should be as many rules as necessary to reduce risks.
1 2 3 4 5

11. Today, most companies in my country cannot compete without special treatment.
1 2 3 4 5

12. Laws exist because somebody in power wants to limit our freedom.
1 2 3 4 5

13. People in my country have lost the talent to invent things like they once did.
1 2 3 4 5

14. No one is really hurt if I copy a CD, tape, or video film.
1 2 3 4 5

15. I feel there is a spirit of teamwork in the department where I work.
1 2 3 4 5

16. I feel there is a spirit of teamwork at the dealership where I work.
1 2 3 4 5

17. I feel like an important member of the department where I work.
1 2 3 4 5

18. I feel like an important member of the dealership where I work.
1 2 3 4 5

19. In this task, we ask you to imagine that you are making a decision that will produce a certain amount of income for you and a certain amount for your **dealership**. You will make your decisions by circling either the letter A, B, or C, producing income for yourself and for the dealership. You will imagine that the dealership is making choices by circling either A, B, or C as well, but you will not see what letters are chosen by the dealership.

Here's an example of how this task works:

	A	B	C
You get	500	500	550
Dealership gets	100	500	300

In this example, if you chose A you would receive 500 points and the dealership would receive 100 points; if you chose B, you would receive 500 points and the dealership 500; and if you chose C, you would receive 550 points and the dealership 300. So, you see that your choice influences both the number of points you receive and the number of points the dealership receives.

Before you begin making choices, please keep in mind that there are no right or wrong answers--choose the option that you, for whatever reason, prefer most. Also, remember that the points have value: The more of them you accumulate, the better for you.

For each of the nine choice situations, circle A, B, or C, depending on which column you prefer most:

		A	B	C
(1)	You get	480	540	480
	Dealership gets	80	280	480
		A	B	C
(2)	You get	560	500	500
	Dealership gets	300	500	100
		A	B	C
(3)	You get	520	520	580
	Dealership gets	520	120	320
		A	B	C
(4)	You get	500	560	490
	Dealership gets	100	300	490
		A	B	C
(5)	You get	560	500	490
	Dealership gets	300	500	90
		A	B	C
(6)	You get	500	500	570
	Dealership gets	500	100	300
		A	B	C
(7)	You get	510	560	510
	Dealership gets	510	300	110
		A	B	C
(8)	You get	550	500	500
	Dealership gets	300	100	500
		A	B	C
(9)	You get	480	490	540
	Dealership gets	100	490	300

20. In this task, we ask you to imagine that you have been randomly paired with a **customer** of your dealership, whom we will refer to simply as "Customer." You will make your decisions by circling either the letter A, B, or C, producing income for yourself and for the customer. You will imagine that the customer is making choices by circling either A, B, or C as well, but you will not see what letters are chosen by the customer.

Here's an example of how this task works:

	A	B	C
You get	500	500	550
Customer gets	100	500	300

In this example, if you chose A you would receive 500 points and the customer would receive 100 points; if you chose B, you would receive 500 points and the customer 500; and if you chose C, you would receive 550 points and the customer 300. So, you see that your choice influences both the number of points you receive and the number of points the customer receives.

Before you begin making choices, please keep in mind that there are no right or wrong answers--choose the option that you, for whatever reason, prefer most. Also, remember that the points have value: The more of them you accumulate, the better for you. Likewise, from the "customer's" point of view, the more points s/he accumulates, the better for him/her.

For each of the nine choice situations, circle A, B, or C, depending on which column you prefer most:

		A	B	C
(1)	You get	480	540	480
	Customer gets	80	280	480
		A	B	C
(2)	You get	560	500	500
	Customer gets	300	500	100
		A	B	C
(3)	You get	520	520	580
	Customer gets	520	120	320
		A	B	C
(4)	You get	500	560	490
	Customer gets	100	300	490
		A	B	C
(5)	You get	560	500	490
	Customer gets	300	500	90
		A	B	C
(6)	You get	500	500	570
	Customer gets	500	100	300
		A	B	C
(7)	You get	510	560	510
	Customer gets	510	300	110
		A	B	C
(8)	You get	550	500	500
	Customer gets	300	100	500
		A	B	C
(9)	You get	480	490	540
	Customer gets	100	490	300

For the final set of questions, please follow the format of questions 4-18 and rate the following statements on a scale of 1 to 5 according to the extent to which you agree or disagree with them.

21. Success is based on survival of the fittest; I am not concerned about the losers.

**Strongly agree**	**Agree**	**undecided**	**disagree**	**disagree strongly**
**1**	**2**	**3**	**4**	**5**

22. For me, what's right is whatever I can get away with.
1 2 3 4 5

23. In today's world, I feel justified in doing anything I can get away with to succeed.
1 2 3 4 5

24. My main purpose in life is getting as many goodies as I can.
1 2 3 4 5

25. Making a lot of money is my most important goal.
1 2 3 4 5

26. I let others worry about higher values; my main concern is with the bottom line.
1 2 3 4 5

27. People who are stupid enough to get ripped off usually deserve it.
1 2 3 4 5

28. Looking out for myself is my top priority.
1 2 3 4 5

29. I tell other people what they want to hear so that they will do what I want them to do.
1 2 3 4 5

30. I would be upset if my success came at someone else's expense.
1 2 3 4 5

31. I often admire a really clever scam.
1 2 3 4 5

32. I make a point of trying not to hurt others in pursuit of my goals.
1 2 3 4 5

33. I enjoy manipulating other people's feelings.
1 2 3 4 5

34. I feel bad if my words or actions cause someone else to feel emotional pain.
1 2 3 4 5

35. Even if I were trying very hard to sell something, I wouldn't lie about it.
1 2 3 4 5

36. Cheating is not justified because it is unfair to others.
1 2 3 4 5
