# Supplementary material for: When Everyone Wins? Exploring Employee and Customer Preferences for No-Haggle Pricing
Source: Front Psychol. 2018 Sep 6;9:1555. doi: 10.3389/fpsyg.2018.01555 (PMC6136271; doi:10.3389/fpsyg.2018.01555)
Supplement: Supplementary file 2 [file Data_Sheet_2.pdf]

Appendix B

Study 1 -- Raw Output with Annotations

Descriptive Statistics

Scale	Included in Table 1?	Item			N	Mean	Std. Deviation	Std. Error	[illegible] Mean: Lower Bound	[illegible] Mean: Upper Bound	Minimum	Maximum
**Attitudes and Values**	**Yes**	**When customers get treated badly by store salespeople, they should expect it; after all, we are all human.**	**V4**	**1**	**19**	**4.95**	**0.229**	**0.053**	**4.84**	**5.06**	**4**	**5**
				**2**	**25**	**4.32**	**0.900**	**0.180**	**3.95**	**4.69**	**1**	**5**
				**3**	**29**	**4.38**	**0.903**	**0.168**	**4.04**	**4.72**	**1**	**5**
				**Total**	**73**	**4.51**	**0.819**	**0.096**	**4.32**	**4.70**	**1**	**5**
Attitudes and Values	No	Most people can rarely be trusted	v5	1	19	4.11	0.937	0.215	3.65	4.56	2	5
				2	24	4.08	0.654	0.133	3.81	4.36	2	5
				3	29	3.72	0.751	0.139	3.44	4.01	2	5
				Total	72	3.94	0.785	0.093	3.76	4.13	2	5
Attitudes and Values	No	Everyone cheats on their taxes, at least a little bit	v6	1	19	3.68	1.376	0.316	3.02	4.35	1	5
				2	24	3.83	0.868	0.177	3.47	4.20	2	5
				3	29	3.38	0.862	0.160	3.05	3.71	1	4
				Total	72	3.61	1.029	0.121	3.37	3.85	1	5
**Attitudes and Values**	**Yes**	**You must think of yourself first if you want to get ahead in life.**	**v7**	**1**	**19**	**4.05**	**0.970**	**0.223**	**3.58**	**4.52**	**2**	**5**
				**2**	**24**	**3.25**	**1.225**	**0.250**	**2.73**	**3.77**	**1**	**5**
				**3**	**29**	**3.28**	**0.996**	**0.185**	**2.90**	**3.65**	**1**	**5**
				**Total**	**72**	**3.47**	**1.113**	**0.131**	**3.21**	**3.73**	**1**	**5**
Attitudes and Values	No	Successes of other people are a threat to one's power in an organization.	v8	1	19	3.95	1.177	0.270	3.38	4.51	2	5
				2	24	3.88	0.992	0.202	3.46	4.29	2	5
				3	28	3.61	1.066	0.201	3.19	4.02	1	5
				Total	71	3.79	1.068	0.127	3.54	4.04	1	5
**Attitudes and Values**	**Yes**	**It is OK to treat groups of people from other countries or cultures differently than we do people like ourselves.**	**v9**	**1**	**19**	**4.74**	**0.562**	**0.129**	**4.47**	**5.01**	**3**	**5**
				**2**	**24**	**4.08**	**0.776**	**0.158**	**3.76**	**4.41**	**2**	**5**
				**3**	**29**	**4.28**	**0.751**	**0.139**	**3.99**	**4.56**	**2**	**5**
				**Total**	**72**	**4.33**	**0.751**	**0.088**	**4.16**	**4.51**	**2**	**5**
Attitudes and Values	No	There should be as many rules as necessary to reduce risks.	v10	1	19	3.32	1.157	0.265	2.76	3.87	1	5
				2	24	3.04	1.160	0.237	2.55	3.53	1	5
				3	29	2.59	0.946	0.176	2.23	2.95	1	4
				Total	72	2.93	1.105	0.130	2.67	3.19	1	5
**Attitudes and Values**	**Yes**	**Today, most companies in my country cannot compete without special treatment.**	**v11**	**1**	**19**	**3.74**	**0.872**	**0.200**	**3.32**	**4.16**	**2**	**5**
				**2**	**24**	**3.79**	**0.884**	**0.180**	**3.42**	**4.16**	**2**	**5**
				**3**	**29**	**3.17**	**0.759**	**0.141**	**2.88**	**3.46**	**2**	**4**
				**Total**	**72**	**3.53**	**0.872**	**0.103**	**3.32**	**3.73**	**2**	**5**
Attitudes and Values	No	Laws exist because somebody in power wants to limit our freedom.	v12	1	19	4.26	1.098	0.252	3.73	4.79	1	5
				2	24	3.96	0.859	0.175	3.60	4.32	1	5
				3	29	3.83	0.539	0.100	3.62	4.03	2	5
				Total	72	3.99	0.831	0.098	3.79	4.18	1	5
Attitudes and Values	No	People in my country have lost the talent to invent things like they once did.	v13	1	19	4.21	1.134	0.260	3.66	4.76	2	5
				2	24	4.04	0.859	0.175	3.68	4.40	2	5
				3	29	3.90	0.817	0.152	3.59	4.21	2	5
				Total	72	4.03	0.919	0.108	3.81	4.24	2	5
Attitudes and Values	No	No one is really hurt if I copy a CD, tape, or video film.	v14	1	19	3.53	1.541	0.353	2.78	4.27	1	5
				2	24	3.50	0.834	0.170	3.15	3.85	2	5
				3	29	3.17	0.928	0.172	2.82	3.53	1	5
				Total	72	3.38	1.093	0.129	3.12	3.63	1	5
N/A	No	I feel there is a spirit of teamwork in the department where I work.	v15	1	19	2.21	1.437	0.330	1.52	2.90	1	5
				2	25	2.00	0.913	0.183	1.62	2.38	1	4
				3	29	2.34	0.897	0.167	2.00	2.69	1	5
				Total	73	2.19	1.063	0.124	1.94	2.44	1	5

N/A	No	I feel there is a spirit of teamwork at the dealership where I work.	v16	1	19	2.47	1.307	0.300	1.84	3.10	1	5
				2	25	2.24	0.970	0.194	1.84	2.64	1	4
				3	29	2.31	0.761	0.141	2.02	2.60	1	5
				Total	73	2.33	0.987	0.115	2.10	2.56	1	5
N/A	No	I feel like an important member of the department where I work.	v17	1	19	2.26	1.522	0.349	1.53	3.00	1	5
				2	25	2.00	0.913	0.183	1.62	2.38	1	4
				3	29	2.00	1.000	0.186	1.62	2.38	1	5
				Total	73	2.07	1.122	0.131	1.81	2.33	1	5
N/A	No	I feel like an important member of the dealership where I work.	v18	1	19	2.32	1.493	0.342	1.60	3.04	1	5
				2	25	2.16	0.898	0.180	1.79	2.53	1	4
				3	29	2.21	0.940	0.175	1.85	2.56	1	5
				Total	73	2.22	1.083	0.127	1.97	2.47	1	5

ANOVA

					Sum of Squares	df	Mean Square	F	Sig.
**Attitudes and Values**	**Yes**	**When customers get treated badly by store salespeople, they should expect it; after all, we are all human.**	**V4**	**Between Groups**	**5.032**	**2**	**2.516**	**4.075**	**0.021**
				**Within Groups**	**43.215**	**70**	**0.617**		
				**Total**	**48.247**	**72**			
Attitudes and Values	No	Most people can rarely be trusted	v5	Between Groups	2.362	2	1.181	1.967	0.148
				Within Groups	41.416	69	0.600		
				Total	43.778	71			
Attitudes and Values	No	Everyone cheats on their taxes, at least a little bit	v6	Between Groups	2.845	2	1.422	1.358	0.264
				Within Groups	72.266	69	1.047		
				Total	75.111	71			
**Attitudes and Values**	**Yes**	**You must think of yourself first if you want to get ahead in life.**	**v7**	**Between Groups**	**8.704**	**2**	**4.352**	**3.790**	**0.027**
				**Within Groups**	**79.240**	**69**	**1.148**		
				**Total**	**87.944**	**71**			
Attitudes and Values	No	Successes of other people are a threat to one’s power in an organization.	v8	Between Groups	1.580	2	0.790	0.687	0.507
				Within Groups	78.251	68	1.151		
				Total	79.831	70			
**Attitudes and Values**	**Yes**	**It is OK to treat groups of people from other countries or cultures differently than we do people like ourselves.**	v9	**Between Groups**	**4.689**	**2**	**2.345**	**4.582**	**0.014**
				Within Groups	35.311	69	0.512		
				Total	40.000	71			
Attitudes and Values	No	There should be as many rules as necessary to reduce risks.	v10	Between Groups	6.555	2	3.277	2.823	0.066
				Within Groups	80.098	69	1.161		
				Total	86.653	71			
**Attitudes and Values**	**Yes**	**Today, most companies in my country cannot compete without special treatment.**	**v11**	**Between Groups**	**6.164**	**2**	**3.082**	**4.451**	**0.015**
				**Within Groups**	**47.780**	**69**	**0.692**		
				**Total**	**53.944**	**71**			
Attitudes and Values	No	Laws exist because somebody in power wants to limit our freedom.	v12	Between Groups	2.206	2	1.103	1.627	0.204
				Within Groups	46.780	69	0.678		
				Total	48.986	71			
Attitudes and Values	No	People in my country have lost the talent to invent things like they once did.	v13	Between Groups	1.139	2	0.569	0.668	0.516
				Within Groups	58.806	69	0.852		
				Total	59.944	71			
Attitudes and Values	No	No one is really hurt if I copy a CD, tape, or video film.	v14	Between Groups	2.000	2	1.000	0.833	0.439
				Within Groups	82.875	69	1.201		
				Total	84.875	71			

N/A	No	I feel there is a spirit of teamwork in the department where I work.	v15	Between Groups	1.605	2	0.803	0.705	0.498
				Within Groups	79.710	70	1.139		
				Total	81.315	72			
N/A	No	I feel there is a spirit of teamwork at the dealership where I work.	v16	Between Groups	0.606	2	0.303	0.305	0.738
				Within Groups	69.504	70	0.993		
				Total	70.110	72			
N/A	No	I feel like an important member of the department where I work.	v17	Between Groups	0.973	2	0.487	0.380	0.685
				Within Groups	89.684	70	1.281		
				Total	90.658	72			
N/A	No	I feel like an important member of the dealership where I work.	v18	Between Groups	0.269	2	0.135	0.112	0.894
				Within Groups	84.224	70	1.203		
				Total	84.493	72			

As noted in the text: "... using the Holm adjustment, the 16 item-specific p values are ordered from lowest to highest and then divided by the inverse-ranking such that (for example) a pre-determined significance value of .05 is translated to .003 (or .05 divided 16) for the item (among the set of 16) with the lowest p-value."

Holm adjustments	t value	unadjusted p value	rank	Adjusted significance threshold (unadjusted x rank)
v9	4.582	0.014	11.000	0.004545455
v11	4.451	0.015	10.000	0.005
v4	4.075	0.021	9.000	0.005555556
v7	3.790	0.027	8.000	0.00625
v10	2.823	0.066	7.000	0.007142857
v5	1.967	0.148	6.000	0.008333333
v12	1.627	0.204	5.000	0.01
v6	1.358	0.264	4.000	0.0125
v14	0.833	0.439	3.000	0.016666667
v8	0.687	0.507	2.000	0.025
v13	0.668	0.516	1.000	0.05

Primary Psychopathy FULL SCALE reporting (as indicated in Fourth Paragraph of Study 1 Results)

. summ ppsych

Variable	Obs	Mean	SD	MIN	MAX
ppsych	71	3.985915	0.50524	2.8125	5

. oneway ppsych dealernumber

Analysis of Variance

Source	SS	df	MS	F	Prob>F
Between-groups	1.34782544	2	0.673913	2.77	0.0695
Within-groups	16.5209026	68	0.242954		
Total	17.868728	70	0.255268		

POST HOC CONTRAST of DEALERSHIP A versus pooling of DEALERSHIPS B AND C **https://stats.idre.ucla.edu/stata/faq/everything-you-always-wanted-to-know-about-contrasts-but-were-afraid-to-ask/**

. contrast {grp 1 -.5 -.5}, effects

Denominator | 68

Contrast	Std. Error	t	P>|t|
.2981472	.1346205	2.21	0.030

Scale	Included in Table 1?	Item							Lower Bound	Upper Bound		
Primary Psychopathy	No	Success is based on survival of the fittest; I am not concerned about the losers.	p21	1	18	3.83	0.924	0.218	3.37	4.29	2	5
				2	24	3.67	0.816	0.167	3.32	4.01	2	5
				3	29	3.48	0.949	0.176	3.12	3.84	2	5
				Total	71	3.63	0.898	0.107	3.42	3.85	2	5

Primary Psychopathy	No	For me, what's right is whatever I can get away with.	p22	1	18	4.56	0.616	0.145	4.25	4.86	3	5
				2	24	4.33	0.702	0.143	4.04	4.63	3	5
				3	29	4.21	0.559	0.104	3.99	4.42	3	5
				Total	71	4.34	0.631	0.075	4.19	4.49	3	5
Primary Psychopathy	Yes	In today's world, I feel justified in doing anything I can get away with to succeed.	p23	1	18	4.61	0.502	0.118	4.36	4.86	4	5
				2	24	4.08	0.881	0.180	3.71	4.46	2	5
				3	29	4.10	0.618	0.115	3.87	4.34	3	5
				Total	71	4.23	0.721	0.086	4.05	4.40	2	5
Primary Psychopathy	Yes	My main purpose in life is getting as many goodies as I can.	p24	1	18	4.50	0.618	0.146	4.19	4.81	3	5
				2	24	4.29	0.751	0.153	3.97	4.61	3	5
				3	29	3.97	0.778	0.145	3.67	4.26	2	5
				Total	71	4.21	0.754	0.090	4.03	4.39	2	5
Primary Psychopathy	No	Making a lot of money is my most important goal.	p25	1	18	3.67	1.283	0.302	3.03	4.30	1	5
				2	24	3.75	0.944	0.193	3.35	4.15	2	5
				3	29	3.45	0.985	0.183	3.07	3.82	1	5
				Total	71	3.61	1.049	0.124	3.36	3.85	1	5
Primary Psychopathy	No	I let others worry about higher values; my main concern is with the bottom line.	p26	1	18	4.00	1.085	0.256	3.46	4.54	1	5
				2	24	3.96	0.859	0.175	3.60	4.32	2	5
				3	29	3.83	0.658	0.122	3.58	4.08	2	5
				Total	71	3.92	0.841	0.100	3.72	4.11	1	5
Primary Psychopathy	Yes	People who are stupid enough to get ripped off usually deserve it.	p27	1	18	4.67	0.594	0.140	4.37	4.96	3	5
				2	24	4.25	0.794	0.162	3.91	4.59	2	5
				3	29	3.72	0.882	0.164	3.39	4.06	2	5
				Total	71	4.14	0.867	0.103	3.94	4.35	2	5
Primary Psychopathy	No	Looking out for myself is my top priority.	p28	1	18	3.67	1.283	0.302	3.03	4.30	1	5
				2	24	3.46	1.103	0.225	2.99	3.92	1	5
				3	29	3.38	0.979	0.182	3.01	3.75	1	5
				Total	71	3.48	1.094	0.130	3.22	3.74	1	5
Primary Psychopathy	No	I tell other people what they want to hear so that they will do what I want them to do.	p29	1	18	4.17	0.707	0.167	3.82	4.52	3	5
				2	24	4.04	0.550	0.112	3.81	4.27	3	5
				3	29	3.86	0.743	0.138	3.58	4.14	2	5
				Total	71	4.00	0.676	0.080	3.84	4.16	2	5
Primary Psychopathy	No	I would be upset if my success came at someone else's expense.	p30(6-score)	1	18	3.78	1.166	0.275	3.20	4.36	1	5
				2	24	3.71	0.955	0.195	3.31	4.11	2	5
				3	29	3.34	0.936	0.174	2.99	3.70	1	5
				Total	71	3.58	1.009	0.120	3.34	3.82	1	5
Primary Psychopathy	No	I often admire a really clever scam.	p31	1	18	4.00	1.188	0.280	3.41	4.59	2	5
				2	24	3.58	1.139	0.232	3.10	4.06	1	5
				3	29	3.83	0.848	0.157	3.51	4.15	2	5
				Total	71	3.79	1.041	0.124	3.54	4.04	1	5
Primary Psychopathy	No	I make a point of trying not to hurt others in pursuit of my goals.	p32(6-score)	1	18	4.44	0.616	0.145	4.14	4.75	3	5
				2	24	4.04	0.751	0.153	3.72	4.36	2	5
				3	29	4.03	0.566	0.105	3.82	4.25	2	5
				Total	71	4.14	0.661	0.078	3.98	4.30	2	5
Primary Psychopathy	No	I enjoy manipulating other people's feelings.	p33	1	18	4.44	0.784	0.185	4.05	4.83	3	5
				2	24	4.29	0.690	0.141	4.00	4.58	3	5
				3	29	4.00	0.707	0.131	3.73	4.27	2	5
				Total	71	4.21	0.735	0.087	4.04	4.39	2	5
Primary Psychopathy	No	I feel bad if my words or actions cause someone else to feel emotional pain.	p34(6-score)	1	18	4.11	1.231	0.290	3.50	4.72	1	5
				2	24	3.83	1.204	0.246	3.32	4.34	1	5
				3	29	4.00	0.964	0.179	3.63	4.37	1	5
				Total	71	3.97	1.108	0.132	3.71	4.23	1	5
Primary Psychopathy	No	Even if I were trying very hard to sell something, I wouldn't lie about it.	p35(6-score)	1	18	4.44	1.042	0.246	3.93	4.96	1	5

				2	24	4.04	1.042	0.213	3.60	4.48	2	5
				3	29	4.34	0.721	0.134	4.07	4.62	2	5
				Total	71	4.27	0.925	0.110	4.05	4.49	1	5
Primary Psychopathy	No	Cheating is not justified because it is unfair to others.	p36(6-score)	1	18	4.50	0.707	0.167	4.15	4.85	3	5
				2	24	4.04	1.160	0.237	3.55	4.53	1	5
				3	29	4.31	0.712	0.132	4.04	4.58	3	5
				Total	71	4.27	0.894	0.106	4.06	4.48	1	5

ANOVA

					Sum of Squares	df	Mean Square	F	Sig.
Primary Psychopathy	No	Success is based on survival of the fittest; I am not	p21	Between Groups	1.404	2	0.702	0.867	0.425
				Within Groups	55.075	68	0.810		
				Total	56.479	70			
Primary Psychopathy	No	For me, what's right is whatever I can get away with.	p22	Between Groups	1.351	2	0.675	1.731	0.185
				Within Groups	26.536	68	0.390		
				Total	27.887	70			
**Primary Psychopathy**	**Yes**	**In today's world, I feel justified in doing anything I can get away with to succeed.**	**p23**	**Between Groups**	**3.594**	**2**	**1.797**	**3.725**	**0.029**
				**Within Groups**	**32.801**	**68**	**0.482**		
				**Total**	**36.394**	**70**			
**Primary Psychopathy**	**Yes**	**My main purpose in life is getting as many goodies as I can.**	**p24**	**Between Groups**	**3.407**	**2**	**1.704**	**3.180**	**0.048**
				**Within Groups**	**36.424**	**68**	**0.536**		
				**Total**	**39.831**	**70**			
Primary Psychopathy	No	Making a lot of money is my most important goal.	p25	Between Groups	1.285	2	0.643	0.578	0.564
				Within Groups	75.672	68	1.113		
				Total	76.958	70			
Primary Psychopathy	No	I let others worry about higher values; my main concern is with the bottom line.	p26	Between Groups	0.397	2	0.198	0.275	0.761
				Within Groups	49.096	68	0.722		
				Total	49.493	70			
**Primary Psychopathy**	**Yes**	**People who are stupid enough to get ripped off usually deserve it.**	**p27**	**Between Groups**	**10.298**	**2**	**5.149**	**8.279**	**0.001**
				**Within Groups**	**42.293**	**68**	**0.622**		
				**Total**	**52.592**	**70**			
Primary Psychopathy	No	Looking out for myself is my top priority.	p28	Between Groups	0.932	2	0.466	0.383	0.683
				Within Groups	82.786	68	1.217		
				Total	83.718	70			
Primary Psychopathy	No	I tell other people what they want to hear so that they will do what I want them to do.	p29	Between Groups	1.093	2	0.547	1.203	0.307
				Within Groups	30.907	68	0.455		
				Total	32.000	70			
Primary Psychopathy	No	I would be upset if my success came at someone else's expense.	p30(6-score)	Between Groups	2.703	2	1.351	1.339	0.269
				Within Groups	68.621	68	1.009		
				Total	71.324	70			
Primary Psychopathy	No	I often admire a really clever scam.	p31	Between Groups	1.860	2	0.930	0.855	0.430
				Within Groups	73.971	68	1.088		
				Total	75.831	70			
Primary Psychopathy	No	I make a point of trying not to hurt others in pursuit of my goals.	p32(6-score)	Between Groups	2.223	2	1.112	2.665	0.077
				Within Groups	28.368	68	0.417		
				Total	30.592	70			
Primary Psychopathy	No	I enjoy manipulating other people's feelings.	p33	Between Groups	2.428	2	1.214	2.332	0.105
				Within Groups	35.403	68	0.521		
				Total	37.831	70			
Primary Psychopathy	No	I feel bad if my words or actions cause someone else to feel emotional pain.	p34(6-score)	Between Groups	0.833	2	0.416	0.333	0.718
				Within Groups	85.111	68	1.252		
				Total	85.944	70			

Scale	Included in Table 1?	Item			Sum of Squares	df	Mean Square	F	Sig.
Primary Psychopathy	No	Even if I were trying very hard to sell something, I wouldn't lie about it.	p35(6-score)	Between Groups	1.961	2	0.980	1.150	0.323
				Within Groups	57.955	68	0.852		
				Total	59.915	70			
Primary Psychopathy	No	Cheating is not justified because it is unfair to others.	p36(6-score)	Between Groups	2.250	2	1.125	1.426	0.247
				Within Groups	53.665	68	0.789		
				Total	55.915	70			

Holm adjustments	t value	unadjusted p value	rank	Adjusted significance threshold (unadjusted x rank)
	8.279	0.001	16	0.003125
	3.725	0.029	15	0.003333333
	3.180	0.048	14	0.003571429
	2.665	0.077	13	0.003846154
	2.332	0.105	12	0.004166667
	1.731	0.185	11	0.004545455
	1.426	0.247	10	0.005
	1.339	0.269	9	0.005555556
	1.203	0.307	8	0.00625
	1.150	0.323	7	0.007142857
	0.867	0.425	6	0.008333333
	0.855	0.430	5	0.01
	0.578	0.564	4	0.0125
	0.383	0.683	3	0.016666667
	0.333	0.718	2	0.025
	0.275	0.761	1	0.05

Descriptive Statistics

Scale	Included in Table 1?	Item			N	Mean	Std. Deviation	Std. Error	95% Confidence Interval for Mean Lower Bound	Upper Bound	Minimum	Maximum
Inclusion of Other in Self - Dealership	No	Inclusion of Other in Self - Dealership	IOSldlr	1	19	4.95	2.345	0.538	3.82	6.08	1	7
				2	25	4.96	1.968	0.394	4.15	5.77	2	7
				3	28	5.00	1.678	0.317	4.35	5.65	2	7
				Total	72	4.97	1.943	0.229	4.52	5.43	1	7
Inclusion of Other in Self - Customer	No	Inclusion of Other in Self - Customer	IOSlcust	1	19	4.84	1.922	0.441	3.92	5.77	1	7
				2	24	5.33	1.494	0.305	4.70	5.96	2	7
				3	28	5.00	1.886	0.356	4.27	5.73	1	7
				Total	71	5.07	1.759	0.209	4.65	5.49	1	7
Inclusion of Other in Self - Employees	No	Inclusion of Other in Self - Employees	IOSlemp	1	19	3.42	2.116	0.486	2.40	4.44	1	7
				2	24	4.21	1.978	0.404	3.37	5.04	1	7
				3	27	4.07	2.018	0.388	3.28	4.87	1	7
				Total	70	3.94	2.028	0.242	3.46	4.43	1	7
**SVO - Prosocial to the Dealership**	**No (Figure 1)**	**SVO - Prosocial to the Dealership**	**Prodlr**	**1**	**18**	**0.94**	**0.236**	**0.056**	**0.83**	**1.06**	**0**	**1**
				**2**	**20**	**0.70**	**0.470**	**0.105**	**0.48**	**0.92**	**0**	**1**
				**3**	**25**	**0.64**	**0.490**	**0.098**	**0.44**	**0.84**	**0**	**1**
				**Total**	**63**	**0.75**	**0.439**	**0.055**	**0.64**	**0.86**	**0**	**1**
**SVO - Prosocial to the Customer**	**No (Figure 1)**	**SVO - Prosocial to the Customer**	**Procust**	**1**	**18**	**0.72**	**0.461**	**0.109**	**0.49**	**0.95**	**0**	**1**
				**2**	**20**	**0.40**	**0.503**	**0.112**	**0.16**	**0.64**	**0**	**1**
				**3**	**26**	**0.65**	**0.485**	**0.095**	**0.46**	**0.85**	**0**	**1**
				**Total**	**64**	**0.59**	**0.495**	**0.062**	**0.47**	**0.72**	**0**	**1**

ANOVA

		Sum of Squares	df	Mean Square	F	Sig.

Inclusion of Other in Self - Dealership	No	Inclusion of Other in Self - Dealership
Inclusion of Other in Self - Customer	No	Inclusion of Other in Self - Customer
Inclusion of Other in Self - Employees	No	Inclusion of Other in Self - Employees
**SVO - Prosocial to the Dealership**	**No (Figure 1)**	**SVO - Prosocial to the Dealership**
**SVO - Prosocial to the Customer**	**No (Figure 1)**	**SVO - Prosocial to the Customer**

IOSIdlr	Between Groups	0.037	2	0.019	0.005	0.995
	Within Groups	267.907	69	3.883		
	Total	267.944	71			
IOSIcust	Between Groups	2.788	2	1.394	0.443	0.644
	Within Groups	213.860	68	3.145		
	Total	216.648	70			
IOSIemp	Between Groups	7.330	2	3.665	0.888	0.416
	Within Groups	276.442	67	4.126		
	Total	283.771	69			
**Prodlr**	**Between Groups**	**1.032**	**2**	**0.516**	**2.839**	**0.066**
	**Within Groups**	**10.904**	**60**	**0.182**		
	**Total**	**11.937**	**62**			
**Procust**	**Between Groups**	**1.142**	**2**	**0.571**	**2.436**	**0.096**
	**Within Groups**	**14.296**	**61**	**0.234**		
	**Total**	**15.438**	**63**			

POST HOC CONTRAST of DEALERSHIP A versus pooling of DEALERSHIPS B AND C https://stats.idre.ucla.edu/stata/faq/everything-you-always-wanted-to-know-about-contrasts-but-were-afraid-to-ask/
immediately after ANOVA comparing all three dealerships regarding SVO - Prosocial to the Dealership

. contrast {dealernumber 1 -.5 -.5}, effects

Denominator | 60

Contrast	Std. Error	t	P>|t|
.2744444	.1191045	2.30	0.025

POST HOC CONTRAST of DEALERSHIP A versus pooling of DEALERSHIPS B AND C https://stats.idre.ucla.edu/stata/faq/everything-you-always-wanted-to-know-about-contrasts-but-were-afraid-to-ask/
immediately after ANOVA comparing all three dealerships regarding SVO-Prosocial to the Customer

. contrast {dealernumber 1 -.5 -.5}, effects // following reg procust i.grp

Denominator | 61

Contrast	Std. Error	t	P>|t|
0.1952991	0.1349172	1.45	0.153

Study 2 -- Raw Output with Annotations

	Mean	N	Std. Devia	Std. Error Mean
	5.1	117	1.567	0.145
**Retail1 (Fixed Pricing)**	3.5	117	1.659	0.153
**Retail2 (Variable Pricing)**				

	N	Correlation	Sig.
	117	-0.119	0.2
Retail1 & Retail2			

	Paired Differences					t	df	Sig. (2-tailed)
	Mean	Std. Deviation	Std. Error	95% Confidence Interval of the Difference				
				Lower	Upper			
Retail1 - Retail2	1.607	2.414	0.223	1.165	2.049	7.2	116	0

T-Test

MEN ONLY

Paired Samples Statistics		Mean	N	SD	SE Mean
	Retail1	4.88	74	1.57	0.182
Pair 1	Retail2	3.72	74	1.601	0.186

Paired Samples Test	Paired Differences					t	df	Sig. (2-tailed)
	Mean	SD	SE Mean	e Interval of the Difference				
				Lower	Upper			

Pair 1	Retail1 - Retail2	1.162	2.146	0.249	0.665	1.659	4.66	73	0

WOMEN ONLY

Paired Samples Statistics		Mean	N	SD	SE Mean
	Retail1	5.49	43	1.502	0.229
Pair 1	Retail2	3.12	43	1.707	0.26

Paired Samples Test		Paired Differences					t	df	Sig. (2-tailed)
		Mean	SD	SE Mean	e Interval of the Difference				
					Lower	Upper			
Pair 1	Retail1 - Retail2	2.372	2.673	0.408	1.55	3.195	5.82	42	0

AGE

reg retail1favor age

**Coefficients**		Unstandardized Coefficients		t	Sig.
**Model**		**B**	**SE**		
	AGE	0.002	0.026	0.091	0.927
	(Constant)	1.588	0.307	5.176	0

a Dependent Variable: Retail1FAVOR

Study 2 preference choices
Retail1 (Fixed Pricing)

3	6	fairness is important in the business world	-3
4	7	The first store seems like a new concept type of thing that may not be a successful business.	-3
4	7	Where negotiation can be obtained then higher wages are assured!.....	-3
3	5	I seems more fair and reflective of supply and demand.	-2
4	6	My friend is extroverted and likes give-and-take of negotiations.	-2
4	6	Variable pricing would potentially mean a greater bargain and thus leading the customer to prevent buyers remorse.	-2
3	5	i think it is fair to do	-2
4	6	Likes to negotiate with people and has fun doing so.	-2
4	6	It seems to be a novel and unique concept to charge individuals based on their negotiation skills, and thus draws my interest.	-2
4	5	Seems more interesting	-1
1	2	N/A	-1
4	5	Seems that the second option would give more flexibility for individual rewards/raises	-1
6	7	The variable has more potential. He likes the negotiation	-1
4	5	There might be a challenge in persuading customers to pay higher prices	-1
5	6	I have friends whom work in sales.	-1
5	6	i think if customer were charged based on the outcome of the negotiations, you'll get more incentives.	-1

4	4	I reported the same interest rate because as long as his pay is the same, why would he care what the customers are charged?	0
4	4	People are entitled to fair priced goods based on the national economy	0
4	4	n/a	0
1	1	I have not interest.	0
4	4	n/a	0
4	4	N/A	0
4	4	n/a	0
1	1	n/a	0
1	1	I didn't	0
6	6	I don't think either one is a bad choice. It can work in the favor of both the organization and the customer in both circumstances.	0
1	1	Well these days there are workers that want more liberalism and fairness in life and work.	0
7	7	n/a	0
4	4	Just general opinion	0
4	4	liked them equally	0
4	4	I didn't.	0
5	5	I did not.	0
4	3	negotiating for prices is stressfull if that's all you do all day at work	1
3	2	Haggling a price with a customer is far more work that a regular sales job and should come with adjusted pay.	1
5	4	It would be less stressful if customers all receive the same price.	1
5	4	Not sure	1
6	5	I think most people would prefer to not have to negotiate with customers if they are paid the same either way.	1
6	4	You don't want to be responsible for negotiations if you are a retail employee. That is a responsibility of a higher level (higher paid) employee.	2
5	3	seems easier	2
5	3	he is an ethical person	2
5	3	I'd rather be able to predict the prices of products I need.	2
6	4	My Best friend is rather shy and wouldn't like the pressure of sales	2
4	2	everyone appreciates fairness	2
5	3	Its less hassle if all prices are uniform.	2
6	4	I don't like haggling	2
6	4	Most of my friends in retail/service industry wouldn't want to put up with the haggling with customers. THey can barely put up with their customers as it is.	2
6	4	The only fact I know about the friend is that he/she wants to work in a retail position. Since the second one involves some evaluation of the salesperson's performance, I assumed that, barring any other information, the friend would consider this job a bit more burdensome or he/she would be subject to greater scrutiny.	2
5	3	I think all sales people should be paid equally.	2
6	4	I suppose my friends would like that best since it would be less stressful.	2

6	4	The top business would be more straight forward to work for. The bottom organization would require a high level of interpersonel skills to be profitable.	2
5	3	The steady uniform prices would be less stressful	2
4	2	Allowing customers to bargain sounds like a pain in the ass for salespersons.	2
4	2	everybody is equal and should be charged the same although I understand that in some situations the price may be lowered depending on the situation. (Somebody that is friendly vs. somebody that is very rude the rude person may be charged the normal price where a nice person may receive a discount just for their bombastic personality) I suppose it's hard for me to judge because I've never been in a market situation where I could barter for better prices.	2
6	4	you have to work harder in the second company	2
6	4	I believe my friend would want everyone to be treated equally.	2
5	2	Uniform is safe where everyone gets paid the same.	3
7	4	negotiation is worthless if commission isn't involved	3
6	3	charging people the same amount would be much simpler and easier	3
6	3	Personally, my best friend has a high value on fairness and probably couldn't negociate well.	3
5	2	fairness	3
7	4	He doesn't seem like a barginer	3
6	3	People shouldn't be treated differently by how they negotiate.	3
7	4	It's better to get payed a static and equal amount.	3
7	4	I would think that dealing with customers who are constantly trying to haggle a better deal would be more unnerving to my friend.	3
6	3	My friend wouldn't like being responsible for how much the customer pays.	3
6	2	Would rather deal with customers who will not try to haggle with you since you are getting paid the same at both locations.	4
6	2	I think the one with uniform prices would require less involved work.	4
6	2	No ones wants to have to deal with different prices for different people.	4
7	3	My friend would not want to have to negotiate with customers about pricing. It would make the job less attractive.	4
7	2	It is more difficult to have to haggle with customers over price	5
7	2	The organization that offers variable pricing that depends on the negotiations with salespeople is less to offer a consistent rate of pay and benefits over time.	5
7	2	fairness	5
7	1	It seems the most fair.	6

7	1	The reason that I picke the first to be my most interest, is simply because when it comes down to a job that negotiates, from my experience, that's a commissions type job, which doesnt always garuntee that you'll make a good income, however the first one, I would know exactly what I make, and i wouldn't have to worry about the stress of trying to constantly sale to people that don't want to pay what it's worth.	6
2	6	I like that the customers have to be nice to get good prices.	-4
4	7	My friend likes to negotiate	-3
5	6	I believe that salespeople should have to work for their earnings as not all are going to act the same to their customers.	-1
4	5	A salesperson with more skill would be paid higher.	-1
2	3	Due to the employee being charged for a uniform,	-1
4	5	the second job might be more interesting	-1
5	5	My interest would be equal in either option.	0
4	4	I think that a friend consider both options equally.	0
4	4	I don't have a preference between the two.	0
4	4	I would be equally interested in both. For me it's about the fair wages.	0
4	4	n/a	0
4	4	NA	0
4	4	changing prices	0
6	5	Not paid depending on sales negotiations	1
5	4	I feel it would be more easier and harmonic to work in an environment with set prices.	1
3	1	I'm not a good negotiator	2
7	5	my best friend is a fair person and would like to work for a company that can be fair to both the employees and the customers.	2
5	3	In the second option you would have to use more persuasion to get a sale.	2
6	4	She is not very good at negotiating, but is great at performing regular work duties	2
7	5	Fairness is always important in retail, so uniform prices would be best	2
6	4	If the customers are charged a uniform price, it's easier to keep track of how many items or units were sold. I think the customers would be treated fairly no matter their status, race, gender or sexual orientation. A uniform price seems direct and transparent.	2
6	4	Negotiations might result in a more volatile workplace, and might be more prone to arguments with customers.	2
5	2	The second option does not seem fair or right. The first seems more equitable, and my best friend is a fair minded person.	3
6	3	It is easier to sale with uniform prices.	3
4	1	My friend would not like that some customers woul dend up paying more or less than others based on who they happen to talk to.	3
7	4	It's better to have a for sure income than to have a varying one, since there is no guarantee that it will stay the same.	3

7	4	I think a stable wage and uniform price is much better than a variable.	3
6	3	My friend has social anxitey and would not enjoy a job of bargaining with customers.	3
4	1	I think all customers should be charged fairly	3
6	2	I cannot stand "haggling" and I think this goes for a lot of people, especially the customers. I would feel like I was making customers uncomfortable in the second scenario.	4
7	3	My best friend is uncomfortable with negotiating.	4
7	2	My friend does not enjoy haggling with people.	5
6	1	I would rather not have to deal with customers trying to bargain for a better price all day.	5
7	2	I think my best friend would much rather work for the organization that has uniform prices, rather than needing to work at a place that the pricing is variable depending on the customer negotiating--I think they would not want to deal with the added hassle of negotiating prices with customers.	5
7	2	The person I used as my 'best friend' doesn't like sales people that 'size you up' or have one person pay more then another for any reason. She hates the negotiating thing.	5
7	1	People shouldn't be charged based on how they dealt with the salespeople, that's crazy.	6
7	1	my friend doesn't like confrontation and wouldn't excel at a job with variable pricing	6
7	1	It seems more fair to have prices based on something more fair than how well you can negotiate with a salesperson. Uniform prices are the most fair.	6
7	1	Because the variable one would be more work.	6
7	1	In the information era, it is easy to find the high and low end price of any item. When a customer discovers he or she has paid a higher amount than another he or she may become quite disgruntled. I do not have a single friend that would enjoy having disgruntled customers returning to them with his or her complaints on pricing. It's an added stress and leads to confrontation unless my friend is selling a vehicle. It seems only acceptable when buying a house or vehicle to expect variable pricing.	6
7	1	Yes I reported retail organization as having more interest because employees are paid in wages which would be better than being paid as a sales person.	6
7	1	Customers should pay equal prices for equal services/goods.	6
7	1	Everyone should be charged the same.	6
